# Dental Care in High‐Risk Pregnancy: A Scoping Review

**DOI:** 10.1111/scd.70246

**Published:** 2026-09-10

**Authors:** Kelly Fernanda Molena, Maya Fernanda Manfrin Arnez, Francisco Wanderley Garcia de Paula‐Silva

**Affiliations:** ^1^ Department of Pediatric Dentistry, School of Dentistry of Ribeirão Preto University of São Paulo Ribeirão Preto Brazil; ^2^ Department of Population Oral Health, Inclusive Care Clinic, University of Illinois Chicago College of Dentistry, University of São Paulo Chicago Illinois USA; ^3^ Department of Dentistry and Oral Medicine, Hospital das Clínicas Ribeirão Preto Medical School, University of São Paulo Ribeirão Preto Brazil

**Keywords:** dental care, high‐risk pregnancy, prenatal care, secondary health care

## Abstract

**Objectives:**

To map evidence on dental care and oral health conditions among women with high‐risk pregnancies, identifying care settings, clinical approaches, and knowledge gaps relevant to oral health practice.

**Methods:**

This scoping review followed PRISMA‐ScR guidelines and was registered in the Open Science Framework. PubMed/MEDLINE, Web of Science, SciELO, LILACS, the Cochrane Library, and Google Scholar were searched without language or publication‐year restrictions. Eligible human studies included pregnant women explicitly classified as high‐risk and addressed oral health conditions or dental care assessed or provided by licensed dentists.

**Results:**

Of 970 records identified, five observational studies met the eligibility criteria. The studies were conducted in Brazil and India and included 1985 high‐risk pregnant women receiving care in primary, specialized outpatient, or hospital settings. Periodontal disease and untreated dental caries were prevalent. One study reported a significant association between periodontitis and preeclampsia. Limited access to dental services, insufficient prenatal dental counseling, and organizational barriers were also identified.

**Conclusion:**

Evidence on dental care in high‐risk pregnancy is limited and heterogeneous and focuses mainly on oral disease prevalence and access to care rather than clinical management protocols. Greater integration of oral health into risk‐stratified prenatal care and research on dental management strategies are needed.

## 1 Introduction

Pregnancy represents a complex and profoundly transformative period in a woman's life, marked by physiological, hormonal, immunological, and social changes that may significantly influence both general and oral health [1]. Although pregnancy is a physiological process, certain maternal clinical conditions can increase the likelihood of adverse maternal and fetal outcomes, characterizing what is defined as a high‐risk pregnancy [2]. In Brazil, public policies such as the *Programa de Humanização do Pré‐Natal e Nascimento* (PHPN), the *Rede Cegonha*, and the *Rede de Atenção Materna e Infantil* (RAMI) underscore the need for multiprofessional follow‐up in this population, highlighting the relevance of incorporating oral health as part of comprehensive prenatal care [3].

The role of the dentist during prenatal care has gained prominence over recent decades, as scientific evidence has increasingly demonstrated the relationship between oral conditions and systemic health, including potential obstetric repercussions [4]. Common gestational oral alterations, such as gingivitis, periodontal disease, dental caries, and dental erosion, may present with greater severity in women with high‐risk pregnancies and have been associated with gestational complications, including preterm birth and low birth weight [4, 5, 6, 7, 8, 9]. Furthermore, systemic conditions such as hypertension, gestational or pre‐existing diabetes mellitus, cardiopathies, systemic infections, hyperemesis gravidarum, and autoimmune disorders can directly influence dental management, requiring individualized protocols and, in some cases, treatment in a hospital setting [10, 11, 12, 13].

Historically, dental care during pregnancy has faced misconceptions and resistance, particularly regarding the safety of dental procedures, medications, and radiographic examinations during gestation [14]. However, recent meta‐analyses and systematic reviews have demonstrated that, when performed using appropriate protocols, dental treatment is both safe and recommended, including restorative, periodontal, and surgical procedures when clinically indicated [15, 16, 17].

Despite the growing literature on oral health during pregnancy, evidence specifically focused on dental care for high‐risk pregnant women remains limited and heterogeneous, hindering the development of consistent clinical guidance. In this context, this study aimed to map and synthesize the available scientific evidence on dental management in high‐risk pregnancy, highlighting oral and systemic considerations, care settings, and gaps relevant to clinical dental practice in maternal health.

## 2 Materials and Methods

### 2.1 Protocol and Registration

This scoping review was registered in the Open Science Framework (OSF) (https://osf.io/h45xm/overview?view_only = e5eeedaf6f084c9a8461c120b70b25b8). The review was conducted in accordance with the Preferred Reporting Items for Systematic Reviews and Meta‐Analyses Extension for Scoping Reviews (PRISMA‐ScR) guidelines [18].

The research question was developed using the PCC framework recommended for scoping reviews. The Population (P) comprised pregnant women classified as having high‐risk pregnancies; the Concept (C) encompassed dental care and oral health–related aspects relevant to high‐risk pregnancy, including clinical management, dental examinations, preventive and therapeutic approaches, pharmacological and radiographic considerations, and clinically relevant oral health conditions assessed by dentists; and the Context (C) included all healthcare settings in which dental care may be provided, such as primary care, specialized outpatient services, and hospital‐based dentistry.

Based on this framework, the guiding research question was formulated as follows: *“What evidence is available in the scientific literature regarding dental care, oral health conditions, and clinically relevant management aspects for high‐risk pregnant women?”*

### 2.2 Eligibility Criteria

Studies were considered eligible for inclusion if they involved human populations and included pregnant women explicitly classified as having high‐risk pregnancies, regardless of maternal age or gestational period. The requirement for explicit classification as a high‐risk pregnancy was established a priori to maintain conceptual consistency and to focus the review on populations formally managed within high‐risk prenatal care pathways. Studies addressing individual medical or obstetric conditions commonly associated with increased pregnancy risk were not considered eligible unless the participants were explicitly identified as having high‐risk pregnancies or were analyzed as a distinct high‐risk subgroup.

Eligible studies were required to address dental care, oral health conditions, or clinically relevant findings assessed or managed by licensed dentists, with clear relevance to the dental management of high‐risk pregnant women. Both observational and interventional study designs were considered eligible, provided they contributed evidence related to dental care delivery, oral health assessment, or clinical decision‐making in the context of high‐risk pregnancy.

Studies were excluded if they addressed high‐risk pregnancy without reference to oral health or dental care, or if they focused exclusively on low‐risk pregnancy or general pregnancy populations without stratification or specific analysis of high‐risk conditions. Studies in which oral health‐related care was provided exclusively by health professionals other than licensed dentists were also excluded. In addition, review articles, opinion papers, editorials, letters to the editor, conference abstracts, and publications without full‐text availability were not considered eligible. No restrictions were applied regarding year of publication or language.

### 2.3 Information Sources and Search Strategy

A comprehensive literature search was conducted in PubMed/MEDLINE, Web of Science, SciELO, LILACS, and the Cochrane Library. Gray literature was searched using Google Scholar. The final search was performed on December 17, 2025, and no restrictions were applied regarding language or year of publication.

The search strategies combined controlled vocabulary, including Medical Subject Headings (MeSH) and Descritores em Ciências da Saúde (DeCS), with free‐text terms related to high‐risk pregnancy, dental care, oral health, and healthcare settings. Boolean operators and database‐specific syntax were adapted for each information source. The fields searched and the complete search strings for each database are presented in Table 1. For Google Scholar, the first 406 results ranked by relevance were screened. All retrieved records were exported to Zotero, where duplicate records were identified and removed before screening.

**TABLE 1:** Search strategy for the database used in this scoping review with MeSH/Decs terms and Boolean operators.

Database	Search
PubMed	(“Pregnancy, High Risk” OR “High‐Risk Pregnancy” OR “High‐Risk Pregnancies” OR “High Risk Pregnancy”OR “Pregnancies, High‐Risk”) AND (Dentistry OR oral OR “Dental care” OR “oral management” OR “oral health” OR Clinical OR intervention OR recommendation OR “management strategies”) AND (“primary health care” OR “primary care”OR outpatient OR “Dental Service, Hospital”)
Web of Science	(“Pregnancy, High Risk” OR “High‐Risk Pregnancy” OR “High‐Risk Pregnancies” OR “High Risk Pregnancy”OR “Pregnancies, High‐Risk”) AND (Dentistry OR oral OR “Dental care” OR “oral management” OR “oral health” OR Clinical OR intervention OR recommendation OR “management strategies”) AND (“primary health care” OR “primary care”OR outpatient OR “Dental Service, Hospital”)
Scielo	(high risk pregnancy) AND (dentistry) OR (dental care) OR (oral health) AND (primary care) OR (hospital dentistry)
Lilacs	“High‐risk pregnancy” AND Dentistry OR oral OR “Dental care” OR “oral management” OR “oral health” OR Clinical OR intervention OR recommendation OR “management strategies” AND “primary health care” OR “primary care”OR outpatient OR “Dental Service, Hospital”
Cochrane	(“high risk pregnancy” OR “high‐risk pregnancy”) AND (dentistry OR “dental care” OR “oral health” OR oral OR dental)
Google Scholar (Gray literature)	(“High‐Risk Pregnancy”) AND (“oral care” OR “Dental care” OR “oral management” OR “oral health”) AND (“primary health care” OR “primary care”OR “Dental Service, Hospital”)

### 2.4 Selection of Sources of Evidence

The study selection process was conducted sequentially in three stages: screening of titles, screening of abstracts, and full‐text assessment, based on predefined eligibility criteria. Studies were included if they addressed dental management, clinical recommendations, oral manifestations, or systemic–oral interactions specifically related to high‐risk pregnancy. Studies focusing exclusively on low‐risk pregnancy or general oral health during pregnancy, without distinction of high‐risk conditions, were excluded. Two reviewers independently screened titles, abstracts, and full‐text articles according to the predefined eligibility criteria. Disagreements were resolved through discussion and consensus, with consultation of a third reviewer when necessary. Zotero software was used to support the organization and screening of the records.

### 2.5 Data Items

The following data were extracted from each included study: author and year of publication, country, study design, population characteristics (sample size and age), type of high‐risk pregnancy condition addressed, healthcare context (primary care, specialized outpatient care, or hospital‐based dentistry), dental interventions described, and key findings relevant to dental management in high‐risk pregnancy. Assumptions and simplifications made by the original authors were recorded when explicitly reported.

## 3 Critical Appraisal of Individual Sources of Evidence

A formal methodological quality or risk‐of‐bias assessment of the included studies was not conducted. The purpose of this scoping review was to map the extent, characteristics, and gaps of the available evidence rather than to evaluate intervention effectiveness, estimate pooled effects, or determine the certainty of evidence. Accordingly, critical appraisal was not considered necessary to answer the review question.

### 3.1 Synthesis of Results

The synthesis of results was conducted using descriptive and thematic analysis. Extracted data were summarized narratively and organized into thematic categories reflecting clinical approaches, types of dental care, safety considerations, healthcare settings, and interdisciplinary practices related to dental management in high‐risk pregnancy. This approach allowed for the identification of patterns, gaps, and areas of concentration within the existing literature.

## 4 Results

**FIGURE 1:**
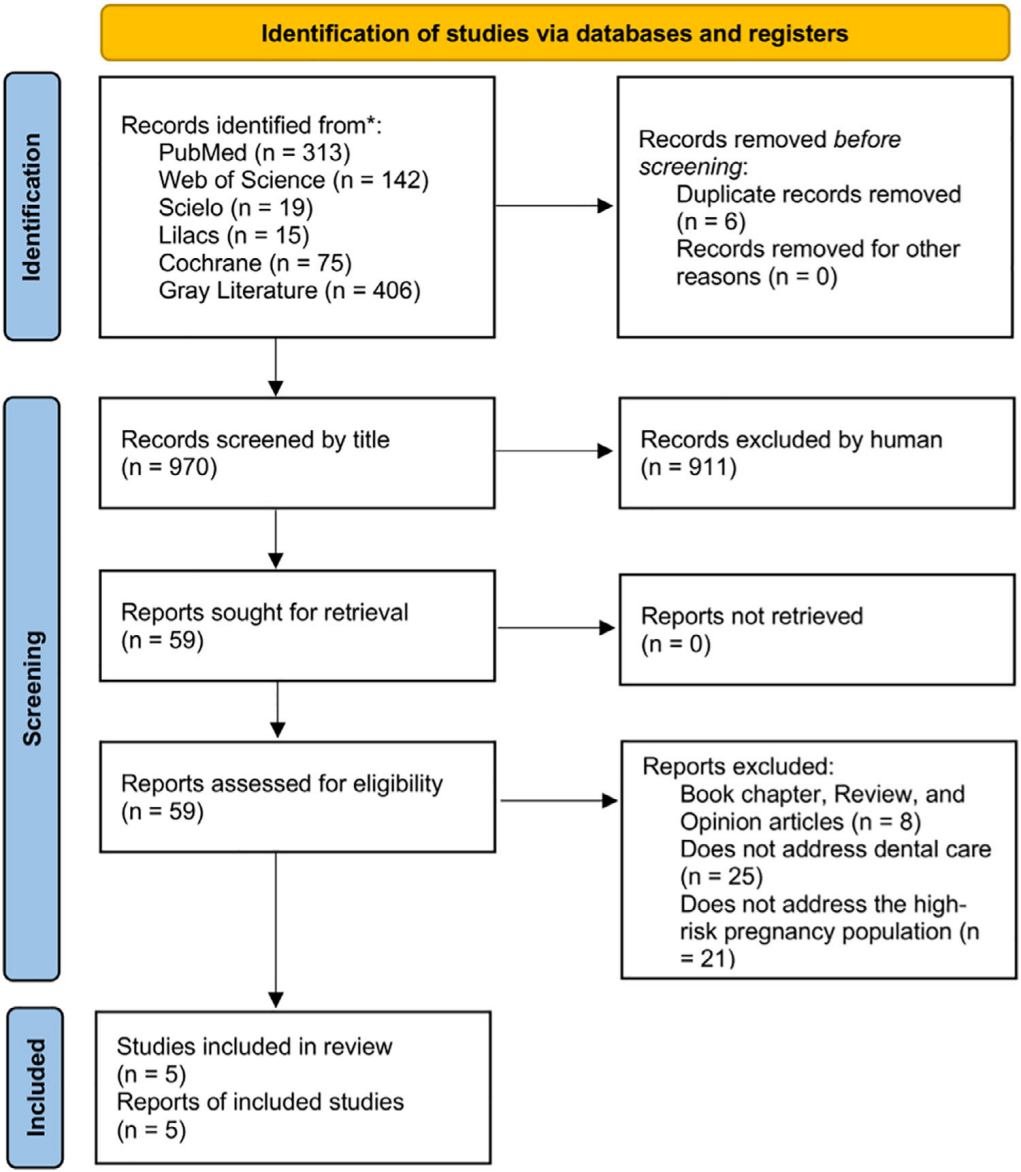
Flowchart of included studies in the scoping review.

The electronic search across the selected databases identified a total of 970 records. After the removal of duplicates, all records underwent title screening. Based on the title assessment, 59 articles were considered potentially relevant and advanced to abstract screening. Following abstract screening, 54 articles were excluded for failing to meet the eligibility criteria, mainly because they did not address oral health or dental care in the context of high‐risk pregnancy, focused mainly on general pregnancy populations, or did not involve dental assessment or management conducted by licensed dentists. As a result, five studies were selected for full‐text assessment. After a comprehensive full‐text review, all five studies met the predefined inclusion criteria and were included in the final synthesis (Figure 1). Reference list screening of the included studies did not identify any additional eligible articles. Therefore, the final sample of this scoping review consisted of five studies.

### 4.1 Characteristics of Included Studies

The five included studies were published between 2010 and 2024 and were conducted in Brazil [6, 19, 20, 21] and India [22]. All studies adopted observational designs, including cross‐sectional approaches [6, 19, 20, 21] and prospective clinical assessments [22].

Altogether, the studies comprised a total of 1985 pregnant women, with a sample size ranging from 76 participants [21] to 1500 high‐risk pregnant women [19], all explicitly classified as having high‐risk pregnancies based on obstetric, clinical, or referral criteria established by each investigation. The conditions defining high‐risk pregnancy varied across studies and included systemic diseases such as hypertension and preeclampsia [22], risk of preterm labor requiring hospital admission [6], and maternal systemic and sociodemographic vulnerability associated with high‐risk prenatal referral [19, 20, 21].

The healthcare settings encompassed primary health care units [21], specialized outpatient prenatal services for high‐risk pregnancy [19, 20], and hospital‐based care, including inpatient services [6, 22] highlighting the diversity of care levels involved in the management of high‐risk gestations.

#### 4.1.1 Data Collection Methods

The included studies employed heterogeneous but complementary data collection strategies, combining structured questionnaires, clinical oral examinations, interviews, and medical record review to assess oral health conditions and dental care among women classified as having high‐risk pregnancies. Questionnaire‐based approaches were primarily used to investigate sociodemographic characteristics, oral health perception, utilization of dental services, and access to prenatal dental care. Lugato et al. [21] applied a structured questionnaire focusing on socioeconomic aspects, self‐reported oral health status, use of dental services, and perception of oral health during pregnancy, with additional questions addressing gestational trimester at data collection and timing of prenatal care initiation. Similarly, Galvan et al. [20] conducted individual face‐to‐face interviews using a structured instrument based on validated tools from the Brazilian Ministry of Health, collecting data on sociodemographic characteristics, gestational information, general and oral health conditions, and guidance to seek dental care during pregnancy.

Clinical oral examinations were conducted in three studies, with emphasis on periodontal status and dental caries. Shetty et al. [22] performed standardized periodontal examinations at enrollment and within 48 h after delivery to evaluate the presence and progression of periodontal disease, assessing gingival index, probing pocket depth, gingival recession, and clinical attachment loss using a UNC‐15 periodontal probe and predefined diagnostic thresholds. Custodio et al. [19] assessed dental status in 1500 high‐risk pregnant women through clinical examinations conducted according to World Health Organization guidelines, using the DMFT index, with sociodemographic and clinical data obtained from medical records and examiner reliability ensured through calibration and kappa statistics. Schievelbein et al. [6] combined medical record review, structured interviews, and comprehensive bedside periodontal examinations in hospitalized women at risk of preterm birth, recording probing pocket depth, bleeding on probing, and clinical attachment level, with periodontal diagnoses established according to the 2018 periodontal disease classification.

#### 4.1.2 Oral Health Conditions and Dental Care in High‐Risk Pregnancy

Across the included studies, oral health assessment was primarily focused on the presence and severity of periodontal disease and dental caries among women with high‐risk pregnancies. Periodontal conditions were clinically evaluated using standardized parameters such as probing pocket depth (PPD), bleeding on probing, gingival index, and clinical attachment loss (CAL) [6, 22]. Gingivitis and periodontitis were highly prevalent in hospitalized women at risk of preterm birth, with extensive gingival inflammation reported in the majority of participants [6].

Dental caries were also frequently reported, often untreated, particularly among high‐risk pregnant women receiving outpatient prenatal care [19]. In addition to clinical findings, some studies addressed oral health behaviors and previous access to dental services as part of the characterization of oral health status [19, 20].

Only one study explicitly investigated the association between oral health conditions and a specific obstetric outcome. Shetty et al. [22] reported a statistically significant association between periodontitis and preeclampsia, identifying periodontal disease as a potential risk indicator among women already classified as having high‐risk pregnancies. In contrast, Schievelbein et al. [6], while documenting a high burden of periodontal inflammation, did not observe a direct association between periodontal disease and the systemic conditions evaluated during hospitalization.

None of the included studies evaluated structured dental management protocols, condition‐specific therapeutic adaptations, pharmacological decision‐making, radiographic practices, or the safety and effectiveness of dental interventions in women with high‐risk pregnancies. Therefore, the available evidence predominantly concerned oral disease burden, oral health perception, and access to care rather than clinical dental management.

### 4.2 Access to Dental Care and Oral Health Perception

Issues related to access to dental care during high‐risk pregnancy were explored in studies conducted in outpatient and primary care settings. Galvan et al. [20] identified limited utilization of dental services during pregnancy, with barriers including lack of guidance from prenatal care teams, insecurity regarding the safety of dental treatment during pregnancy, and organizational challenges within healthcare services.

Perception of oral health and awareness of the importance of dental care during pregnancy were specifically addressed in Lugato et al. [21]. Although most participants classified their oral health as good or regular, a substantial proportion reported not having received guidance regarding dental treatment during pregnancy. Pregnant women who did not perceive oral care as important during gestation were more likely to report poor self‐rated oral health. Across studies, the absence of prenatal dental counseling was consistently associated with reduced engagement with dental services.

Table 2 synthesizes the findings of the included studies.

**TABLE 2:** Overview of observational studies included in this scoping review, presenting oral health conditions, access to dental care, and healthcare settings among women classified as having high‐risk pregnancies.

Reference	Country	Study design	Population, age	High‐risk pregnancy condition	Healthcare context	Dental interventions	Key findings
Lugato et al. [21]	Brazil	Cross‐sectional observational study	76 high‐risk pregnant women, 20 years old	High‐risk pregnancy (clinical/obstetric risk criteria)	Primary care dental services	Oral health perception assessment; guidance during prenatal dental care	Most women recognized the importance of oral care during pregnancy, but lack of guidance was associated with poor self‐perception of oral health, highlighting the need for structured prenatal dental counseling.
Shetty et al. [22]	India	Prospective cohort study	130 pregnant women (26–32 weeks gestation), 26.8 years old	Preeclampsia	Hospital‐based obstetric and dental care	Periodontal examination and diagnosis	Periodontitis was significantly associated with preeclampsia, suggesting periodontal evaluation as a relevant component of care in high‐risk pregnancies.
Custodio et al. [19]	Brazil	Cross‐sectional study	1500 high‐risk pregnant women, 71.4% between 16–34 years old	Arterial hypertension, gestational arterial hypertension, diabetes mellitus, gestational diabetes mellitus, smoking, obesity	Specialized outpatient care	Dental caries assessment	High prevalence of dental caries was observed among high‐risk pregnant women, reinforcing the need for preventive and restorative dental care during prenatal follow‐up.
Schievelbein et al. [6]	Brazil	Cross‐sectional study	89 hospitalized women at risk of preterm labor, 57.3% ≤ 24 years old	Preeclampsia, gestational diabetes, diabetes, hypertension, asthma, anemia, HIV and HPV, syphilis, hypothyroidism, toxoplasmosis	Hospital‐based care	Periodontal examination	High prevalence of gingival inflammation and periodontitis was observed, emphasizing the importance of dental assessment even in hospitalized high‐risk pregnancies.
Galvan et al. [20]	Brazil	Cross‐sectional observational study	190 high‐risk pregnant women, 32 years old	Hypertension, drugs addictive, cardiac diseases, pneumonie, nephropaties, diabetes, gestational diabetes, hemopathies, ginecological malformation, infection diseases, epilepsy, twin pregnancy	Specialized outpatient care	Evaluation of access to and search for dental care	Lack of professional guidance significantly reduced the likelihood of seeking dental care, highlighting organizational and educational barriers to prenatal dental care in high‐risk pregnancies.

## 5 Discussion

This scoping review mapped the available evidence on oral health conditions and dental care in women classified as having high‐risk pregnancies. Despite the clinical relevance and epidemiological magnitude of high‐risk pregnancy, only five observational studies met the inclusion criteria, underscoring that this population remains largely overlooked in dental research. This finding is particularly concerning given that high‐risk pregnancy is defined as a condition in which maternal, fetal, and neonatal health have a higher probability of compromise than in the general population [23], and it may require differentiated clinical protocols, intensified monitoring, and coordinated multiprofessional care across healthcare levels [24, 25].

The included studies demonstrated a high burden of oral disease among women in high‐risk prenatal follow‐up, particularly periodontal inflammation and untreated dental caries [6, 19, 22]. These findings align with established biological mechanisms linking pregnancy‐related hormonal, immunological, and vascular changes to increased susceptibility to gingivitis and periodontitis, which may be further exacerbated by systemic conditions commonly present in high‐risk pregnancies, such as diabetes mellitus, hypertensive disorders, cardiovascular disease, and immune dysregulation [4, 5, 6, 7, 8, 9, 26]. The high prevalence of gingival inflammation observed in hospitalized women at risk of preterm birth reinforces the need for routine dental care assessment even in acute or inpatient obstetric settings [6].

Only one study explicitly explored the association between oral disease and a specific adverse obstetric outcome within a high‐risk population. Shetty et al. [22] identified a significant association between periodontitis and preeclampsia, supporting the hypothesis that periodontal inflammation may act as a modifiable risk indicator in pregnancies already burdened by systemic vulnerability. These findings are biologically plausible and consistent with proposed mechanisms involving systemic dissemination of periodontal pathogens or inflammatory mediators, endothelial dysfunction, and placental inflammation [8, 27, 28]. Conversely, Schievelbein et al. [6] did not observe associations between periodontal disease and the systemic conditions assessed during hospitalization, despite documenting extensive periodontal inflammation. Rather than contradicting the potential relevance of oral health, this divergence highlights the heterogeneity inherent to high‐risk pregnancy phenotypes and the limitations of cross‐sectional designs in capturing dynamic systemic–oral interactions during pregnancy [29].

Beyond clinical findings, this review also highlights persistent barriers to dental care access and utilization among high‐risk pregnant women. Studies conducted in outpatient and primary care contexts reported low use of dental services, insufficient guidance from prenatal care teams, and insecurity regarding the safety of dental treatment during pregnancy [20, 21]. These barriers are particularly problematic in high‐risk pregnancy, where women are already highly engaged with healthcare services and where opportunities for preventive counseling and referral should be maximized. From a health systems perspective, these findings suggest failures in integrating dental care into structured prenatal pathways, despite national and international recommendations advocating multiprofessional and risk‐stratified care models [24, 25].

Although the original review question included dental management and clinically relevant decision‐making, the available evidence was largely limited to oral disease prevalence, oral health perception, and access to dental services. None of the included studies evaluated structured dental care protocols, therapeutic adaptations according to specific systemic conditions, pharmacological considerations, radiographic decision‐making, or the safety and effectiveness of dental interventions in high‐risk pregnancy. Therefore, this review cannot establish evidence‐based clinical management recommendations for this population. Rather, the absence of such studies represents one of the principal gaps identified by the review and underscores the need for prospective clinical research focused on individualized and interdisciplinary dental care.

The findings of this scoping review reinforce that oral health care should be considered an integral component of high‐risk prenatal care [6, 19, 22]. Periodontal disease, dental caries, and other oral conditions such as dental erosion, particularly in women with hyperemesis gravidarum or gastroesophageal reflux, can negatively impact maternal quality of life and potentially exacerbate systemic inflammation [30, 31]. Evidence‐based dental management during pregnancy, including preventive care, periodontal therapy, restorative procedures, and emergency interventions, is safe when performed under appropriate protocols and may contribute to overall maternal stability [15, 32]. Moreover, multiprofessional collaboration between dentists, obstetricians, nurses, and other specialists is essential to ensure coordinated, safe, and individualized care for this vulnerable population [33, 34].

Clinically, the results highlight the need to institutionalize prenatal dental care within high‐risk pregnancy pathways, incorporating routine oral screening, structured counseling, and clear referral mechanisms across primary, specialized outpatient, and hospital‐based services. Strengthening oral health literacy and education during high‐risk prenatal follow‐up may improve self‐care behaviors, reduce disease burden, and enhance adherence to dental services, particularly among socially and clinically vulnerable women [35, 36, 37].

This scoping review has several limitations that should be acknowledged. The evidence base was limited to five observational studies, predominantly cross‐sectional, which restricted causal inference and precluded evaluation of the effectiveness of dental interventions in improving obstetric outcomes. Definitions of high‐risk pregnancy varied across studies, and oral health outcomes were heterogeneous, with a predominant focus on periodontal disease and dental caries and limited investigation of clinical management strategies. This heterogeneity also reflects inconsistencies in how high‐risk pregnancy is operationalized in the literature, which may affect the identification and inclusion of relevant studies in evidence syntheses. No formal methodological quality or risk‐of‐bias assessment was performed. Although this approach was consistent with the mapping purpose of the review, it limits the ability to determine the methodological robustness of the individual studies and should be considered when interpreting the findings.

An additional limitation concerns the operational definition adopted in this review, which required participants to be explicitly classified as having high‐risk pregnancies or analyzed as a distinct high‐risk subgroup. This criterion was established a priori to maintain conceptual consistency and to focus the synthesis on women formally managed within high‐risk prenatal care pathways. However, it may have excluded relevant studies investigating individual conditions commonly associated with increased obstetric risk, including gestational diabetes, hypertensive disorders, preeclampsia, cardiovascular disease, and autoimmune disorders, when the study population was not explicitly described as high‐risk. Consequently, the mapped evidence should not be interpreted as representing the entire body of literature on oral health in women with pregnancy‐related systemic or obstetric risk conditions. Finally, because most included studies were conducted in Brazil, the generalizability of the findings to other healthcare systems may be limited.

Nonetheless, this scoping review provides a focused and clinically relevant synthesis of studies that explicitly address high‐risk pregnancy as a distinct category within prenatal care, highlighting consistent patterns of high oral disease burden, limited access to dental care, and lack of structured integration of oral health within high‐risk prenatal services.

In conclusion, this scoping review demonstrates that high‐risk pregnant women remain an underrepresented population in dental research, despite consistently high levels of oral disease and documented barriers to care. The limited and heterogeneous evidence underscores the urgent need for well‐designed studies focusing on dental management protocols, interdisciplinary care models, and preventive strategies tailored to high‐risk pregnancy. Integrating oral health into structured, risk‐stratified prenatal care is essential to promote maternal–fetal health and reduce avoidable morbidity in this clinically complex population.

## 6 Conclusion

This scoping review shows that women explicitly classified as having high‐risk pregnancies remain underrepresented in dental research. The available studies predominantly address periodontal disease, dental caries, oral health perception, and barriers to dental care, while evidence concerning specific management protocols, therapeutic strategies, pharmacological considerations, and clinical decision‐making remains absent. These findings highlight the need for prospective studies evaluating safe, individualized, and interdisciplinary dental care approaches for different high‐risk pregnancy conditions. Greater integration of oral health assessment, counseling, and referral into risk‐stratified prenatal pathways is also needed.

## Funding

The authors thank the Conselho Nacional de Desenvolvimento Científico e Tecnológico, CNPq. (103335/2025‐0) for all support.

The Article Processing Charge for the publication of this research was funded by the Coordenaçáo de Aperfeiçoamento de Pessoal de Nível Superior ‐ Brasil (CAPES) (ROR identifier: 00x0ma614).

## Conflicts of Interest

The author declares no conflicts of interest.

## References

[1] E. P. Davis and A. J. Narayan , “Pregnancy as a Period of Risk, Adaptation, and Resilience for Mothers and Infants,” Developmental Psychopathology 32, no. 5 (2020): 1625–1639. 10.1017/S0954579420001121 PMC7863987 33427164

[2] National Academies of Sciences, Engineering, and Medicine E. P. Backes and S. C. Scrimshaw , eds., “Epidemiology of Clinical Risks in Pregnancy and Childbirth,” in Birth Settings in America: Outcomes, Quality, Access, and Choice (National Academies Press, 2020). 32049472

[3] P. K. Mortelaro , J. F. Cirelli , N. Z. Narchi , and E. A. Campos , “From the ‘Rede Cegonha’ to ‘RAMI’: Tensions Between Paradigms of Maternal and Infant Health Care,” Saude Debate 48, no. 140 (2024): e8152.

[4] F. W. G. Paula‐Silva , A. A. Oliveira , A. C. Freitas , and M. F. M. Arnez , “Pre‐natal Odontologico [Dental Prenatal Care],” in Pré‐natal Odontológico [Dental Prenatal Care], ed. F. W. G. Paula‐Silva , (Santos Publicações, 2023), 3–24.

[5] A. Shahi , S. Khosravi , F. Rezvan , A. Salehi , M. B. Mahmoudi , and A. Amiri , “Evaluation of the Periodontal Disease on Oral Microorganisms During Pregnancy: A Systematic Review and Meta‐Analysis,” Journal of Clinical and Translational Research 9, no. 3 (2023): 144–152. 37181818 PMC10171318

[6] B. S. Schievelbein , R. P. Casarin , M. S. da Mota Kruger , et al., “Systemic Profile and Periodontal Condition of Hospitalized Women With High‐Risk Pregnancy: A Cross‐Sectional Study,” Maternal and Child Health Journal 27, no. 7 (2023): 1264–1271. 37004625 10.1007/s10995-023-03659-8

[7] M. P. Pecci‐Lloret , C. Linares‐Pérez , M. R. Pecci‐Lloret , F. J. Rodríguez‐Lozano , and O.‐S. RE , “Oral Manifestations in Pregnant Women: A Systematic Review,” Journal of Clinical Medicine 13, no. 3 (2024): 707. 38337401 10.3390/jcm13030707 PMC10856094

[8] M. Zhao , H. Chang , Y. Yue , X. Zeng , S. Wu , and X. Ren , “The Association Between Periodontal Disease and Adverse Pregnancy Outcomes: A Bibliometric Analysis From 2000 to 2023,” Frontiers in Medicine 12 (2025): 1526406. 39906598 10.3389/fmed.2025.1526406 PMC11790436

[9] H. Xu , M. Cai , H. Xu , X. J. Shen , and J. Liu , “Role of Periodontal Treatment in Pregnancy Gingivitis and Adverse Outcomes: A Systematic Review and Meta‐Analysis,” Journal of Maternal‐Fetal & Neonatal Medicine 38, no. 1 (2025): 2416595. 39721768 10.1080/14767058.2024.2416595

[10] J. B. Franco , “Manejo Odontológico Da Gestante De Alto Risco [Dental Management of High‐Risk Pregnant Women],” in Odontologia Hospitalar [Hospital Dentistry], ed. Eduardo FP , Bezinelli LM , and Corrêa L , (Manole, 2019), 253.

[11] P. M. C. Freitas , N. C. Duarte Neto , D. A. Santos , et al., “Urinary Tract Infection in Pregnant Women: Possible Causes,” Brazilian Journal of Implantology and Health Sciences 5, no. 3 (2023): 270–283.

[12] J. M. García‐Martos , F. J. Valverde‐Bolívar , M. T. Campillo‐López , and M. Delgado‐Rodríguez , “Association Between Periodontal Disease and Gestational Diabetes: Systematic Review and Meta‐Analysis,” Primary Care Diabetes 19, no. 1 (2025): 1–6. 39627087 10.1016/j.pcd.2024.11.003

[13] E. S. Vadakekut and H. Mahdy , “Hyperemesis Gravidarum,” in StatPearls [Internet] (StatPearls Publishing, 2026). 30422512

[14] A. Tenenbaum and S. Azogui‐Levy , “Oral Health Knowledge, Attitudes, Practices, and Literacy of Pregnant Women: A Scoping Review,” Oral Health & Preventive Dentistry 21 (2023): 185–198. 37195335 10.3290/j.ohpd.b4100965 PMC11619840

[15] Z. Yenen and T. Ataçağ , “Oral Care in Pregnancy,” Journal of the Turkish‐German Gynecological Association 20, no. 4 (2019): 264–268. 30556662 10.4274/jtgga.galenos.2018.2018.0139 PMC6883753

[16] H. Jang , A. Patoine , T. T. Wu , D. A. Castillo , and J. Xiao , “Oral Microflora and Pregnancy: A Systematic Review and Meta‐Analysis,” Scientific Reports 11, no. 1 (2021): 16870. 34413437 10.1038/s41598-021-96495-1 PMC8377136

[17] U. S. Bhadauria , S. Singh , P. Paswan , P. Bhattacharya , B. Purohit , and H. Priya , “Assessment of the Quality of Guidelines on Oral Health Care During Pregnancy: A Systematic Review,” Evidence‐Based Dentistry 25, no. 4 (2024): 214–215. 10.1038/s41432-024-01046-y 39134686

[18] A. C. Tricco , E. Lillie , W. Zarin , et al., “PRISMA Extension for Scoping Reviews (PRISMA‐ScR): Checklist and Explanation,” Annals of Internal Medicine 169, no. 7 (2018): 467–473. 30178033 10.7326/M18-0850

[19] M. de Custodio LB , T. A. Saliba , O. Saliba , N. A. Saliba , and S. A. S. Moimaz , “Dental Caries Status in High‐Risk Pregnant Women: Systemic Conditions and Sociodemographic Factors,” Revista Valore 6 (2021): e6005.

[20] J. Galvan , D. Bordin , C. B. Fadel , and F. B. T. Alves , “Factors Related to Orientation of Search for Dental Care in High‐Risk Pregnancy,” Revista Brasileira de Saúde Materno Infantil 21, no. 4 (2021): 1143–1153.

[21] V. P. M. Lugato , F. M. Flório , and L. Z. de Souza , “To Evaluate High‐Risk Pregnant Women's Oral Health Perception and Associated Factors,” Revista Brasileira de Saúde Materno Infantil 24 (2024): e20230182.

[22] M. Shetty , P. K. Shetty , A. Ramesh , B. Thomas , S. Prabhu , and A. Rao , “Periodontal Disease in Pregnancy Is a Risk Factor for Preeclampsia,” Acta Obstetricia et Gynecologica Scandinavica 89, no. 5 (2010): 718–721. 20196677 10.3109/00016341003623738

[23] R. Caldeyro Barcia , S. Pose , J. Poseiro , and C. Méndez Bauer , Frecuencia Cardíaca y Equilibrio Acido Base Del Feto [Fetal Heart Rate and Acid‐Base Balance] (Centro Latinoamericano de Perinatología y Desarrollo Humano, 1973).

[24] Brasil. Ministério da Saúde Gestação de Alto Risco: Manual Técnico [High‐Risk Pregnancy: Technical Manual] (Ministério da Saúde, 2010).

[25] Brasil. Ministério da Saúde Manual de Gestação de Alto Risco [High‐Risk Pregnancy Manual] (Ministério da Saúde, 2022).

[26] M. Kashetty , S. Kumbhar , S. Patil , and P. Patil , “Oral Hygiene Status, Gingival Status, Periodontal Status, and Treatment Needs Among Pregnant and Nonpregnant Women: A Comparative Study,” Journal of Indian Society of Periodontology 22, no. 2 (2018): 164–170. 29769772 10.4103/jisp.jisp_319_17 PMC5939025

[27] Y. A. Bobetsis , M. Ide , M. Gürsoy , and P. N. Madianos , “Periodontal Diseases and Adverse Pregnancy Outcomes: Present and Future,” Periodontology 2000 92, no. 1 (2023): 76–88, 10.1111/prd.12486. 37149740

[28] M. A. Ortega , O. Fraile‐Martínez , C. García‐Montero , et al., “The Pivotal Role of the Placenta in Normal and Pathological Pregnancies: A Focus on Preeclampsia, Fetal Growth Restriction, and Maternal Chronic Venous Disease,” Cells 11, no. 3 (2022): 568. 35159377 10.3390/cells11030568 PMC8833914

[29] L. Mendes , B. Emmanuelli , M. C. Segatto , J. K. Knorst , F. Tomazoni , and G. Araújo , “Determinantes do Uso de Serviços Odontológicos no Período Pré‐gestacional e Gestacional: Um Estudo Transversal [Determinants of the use of Dental Services in the Pre‐Gestational and Gestational Periods: A Cross‐Sectional Study],” Cadernos de Saúde Pública 41, no. 7 (2025): e00179524. 40699100 10.1590/0102-311XPT179524 PMC12337696

[30] N. A. B. Islam and A. Haque , “Pregnancy‐Related Dental Problems: A Review,” Heliyon 10, no. 3 (2024): e24259. 38322854 10.1016/j.heliyon.2024.e24259 PMC10845246

[31] X. C. Men , X. P. Du , and Y. Ji , “Effects of Personalized Oral Hygiene Management on Oral Health Status of Pregnant Women,” World Journal of Clinical Cases 12, no. 21 (2024): 4566–4573. 39070809 10.12998/wjcc.v12.i21.4566 PMC11235517

[32] Y. G. M. da Cunha , E. F. da Silva , G. M. Oliveira , C. F. Santos , and A. M. Calvo , “The Use of Different Local Anesthetics in Pregnant Women in Dentistry: A Systematic Review,” Current Reviews in Clinical and Experimental Pharmacology 20 (2025): 133–146. 10.2174/0127724328349965250407082245 40264307

[33] F. M. Baeder , P. F. L. Corazza , A. C. L. Albuquerque , et al., “Perception of the Multidisciplinary Team on the Importance of Dentistry in Primary Care for Pregnant Women in Brazil: Integrative Review,” Research, Society and Development 11, no. 17 (2022): e75111738763.

[34] M. Ebinghaus , C. J. Agricola , J. Schmittinger , N. Makarova , and B. C. Zyriax , “Assessment of Women's Needs and Wishes regarding Interprofessional Guidance on Oral Health in Pregnancy: A Qualitative Study,” BMC Pregnancy Childbirth 24, no. 1 (2024): 471. 38992618 10.1186/s12884-024-06675-w PMC11238511

[35] Y. M. Kamalabadi , M. K. Campbell , N. M. Zitoun , and A. Jessani , “Unfavourable Beliefs About Oral Health and Safety of Dental Care During Pregnancy: A Systematic Review,” BMC Oral Health 23, no. 1 (2023): 762. 37840149 10.1186/s12903-023-03439-4 PMC10577919

[36] M. F. M. Arnez , A. M. Queiroz , A. C. Silva‐Sousa , A. De Rossi , and F. W. G. Paula‐Silva , “Cuidados no Atendimento Odontologico a Gestante [Dental Care for Pregnant Women],” in Pré‐natal Odontológico [Dental Prenatal Care], ed. F. W. G. Paula‐Silva , (Santos Publicações, 2023), 53–90.

[37] M. L. Vera‐Carpio , K. M. Carranza‐Samanez , and J. A. Dulanto‐Vargas , “Myths About Oral Health and Associated Factors in Pregnant Women in a Public Hospital in Peru,” Oral Health & Preventive Dentistry 23 (2025): 123–134. 39973808 10.3290/j.ohpd.c_1845 PMC11880829

